# Exploring undergraduate nursing students’ preparation to care for children and their families in Australia: a scoping review

**DOI:** 10.11124/JBIES-25-00024

**Published:** 2026-10-02

**Authors:** Melisa Jayne Young, Huaqiong Zhou, Yunia Eka Sari Kolikiana, Janie Alison Brown

**Affiliations:** 1 Curtin School of Nursing, Curtin University, Perth, WA, Australia; 2 JBI Evidence Informed Health Care Curtin University, Perth, WA, Australia; 3 Perth Children’s Hospital, Perth, WA, Australia; 4 St John of God Midland Public and Private Hospitals, Perth, WA, Australia

**Keywords:** education, nursing, pediatric

## Abstract

**Objective:**

This scoping review identifies and maps the breadth of evidence describing the preparation of preregistration nursing students to provide care to infants, children, young people, and their families in an Australian context.

**Introduction:**

Nursing graduates in Australia are expected to deliver safe and competent care to children and families across diverse health care settings. However, pediatric nursing education is commonly embedded within integrated lifespan curricula, with limited access to dedicated pediatric units or clinical placements. This lack of structured pediatric preparation has been associated with student anxiety, reduced confidence, challenges in age-appropriate communication, and reluctance to pursue pediatric nursing pathways following graduation, raising concerns about patient safety and workforce sustainability.

**Eligibility criteria:**

This scoping review included published and unpublished literature relevant to undergraduate preregistration nursing students in Australia. Evidence sources published from 2010 onward were eligible and included empirical studies, non-empirical literature, policy documents, professional standards, and gray literature related to the preparation of nursing students to care for children and their families across all health care settings.

**Methods:**

This review followed the JBI methodology for scoping reviews. A 3-step search strategy was used between December 2023 and February 2024 to identify relevant literature across the CINAHL (EBSCOhost), MEDLINE (Ovid), Scopus, and ERIC (ProQuest) electronic databases, in advanced Google searches, and across websites of Australian professional organizations, government bodies, pediatric hospitals, and universities. Two reviewers independently screened citations, extracted data, and synthesized findings. Results were organized according to regulatory requirements and recommendations, educational preparation approaches, and student experiences.

**Results:**

In total, 10 peer-reviewed publications and 29 gray literature sources met the eligibility criteria. According to these sources, efforts to prepare nursing students to care for children commonly involved integrated curriculum approaches, simulation-based learning, and supported work-integrated learning experiences. High-fidelity simulation and alternative placement models to improve knowledge, confidence, communication skills, and clinical reasoning were identified. Immersive experiential learning to create interest in pediatric nursing pathways—particularly among students with limited prior exposure to children, including male students—was investigated. Publicly available information from universities and pediatric hospitals demonstrated variability in preparation requirements and limited transparency regarding pre-placement preparation.

**Conclusions:**

Despite clear professional and regulatory recommendations, the preparation of preregistration nursing students to care for children and families in Australia is inconsistent. Integrating pediatric education across undergraduate curricula, supported by simulation and alternative experiential learning opportunities, may enhance student confidence, readiness, and workforce participation in pediatric nursing.

**Review registration:**

OSF https://osf.io/4mtf6/overview

## Introduction

Australian nursing graduates are required to deliver safe and proficient care to pediatric patients across diverse health care settings.^1^ The transition of nursing education from hospital-based training to higher education institutions in 1974, culminating in a nationwide shift in 1984, marked a departure from the Nightingale system toward a comprehensive program.^2^ This shift aimed to equip graduates with the skills to practice beginner-level nursing across various domains, including general, mental health, disability, pediatric, aged care, and community nursing.

University nursing curricula shifted in response to rapid changes within health care, with a move to pedagogy grounded in educational theories and research that value critical thinking and problem solving over memorization.^3^ Pediatric nursing knowledge and skills are most often integrated within subjects or units across the nursing curriculum, with few universities providing discrete or standalone subjects or units. However, integrating pediatric theory and skills throughout the curriculum can leave nursing students feeling ill-prepared for clinical placements and the pediatric workforce.^4^ The lack of pediatric and maternity clinical placements available to universities for nursing students,^4^,^5^ and the absence of specific requirements for such, contributes to graduates’ lack of age-appropriate communication skills,^6^ which impacts patient safety, as ineffective communication is a leading cause of patient harm.^1^

Nursing students who attend a pediatric placement face significant challenges. A 2023 study by Gibson-Young *et al*.^7^ confirmed previous findings that identified increased nursing student anxiety when caring for unwell children and interacting with their parents. For example, administering medications to pediatric patients requires not only a high degree of precision and skill, but is also coupled with the challenge of ensuring that children cooperate during administration, while at the same time, nursing students are learning in the presence of parents. When faced with this challenge, nursing students may struggle to develop confidence or appear credible to parents and caregivers.^8^ These difficulties can exacerbate feelings of inadequacy and stress, affecting nursing students’ confidence and performance in pediatric nursing settings.^9^

Globally, there is an unparalleled shortage of nurses.^10^ While many nurses enter the profession, retention is poor due to a range of issues including, but not limited to, burnout. Grave concerns of burnout in the pediatric nursing workforce in the US were reported by Sonney and Peck,^11^ who specifically discuss the increase in health care demands following the COVID-19 pandemic. Nursing students’ limited entry into the pediatric workforce after graduation has been attributed to the trend across nursing programs of reducing pediatric undergraduate (UG) curricular content.^12^ In Australia, a national nursing workforce strategy consultation is underway, and while there is a paucity of recent Australian evidence related to the pediatric nursing workforce, concerns have spurred workforce shortage debates in the past,^13^,^14^ with reports indicating a decline in the number of graduates entering pediatric nursing, despite rising cases of children with complex health care needs.^15^,^16^ Betz^15^ attributed this decline to inadequate exposure to pediatric health care during training, outdated teaching methods, and inexperienced faculty.

The transition from student to graduate nurse, termed *work readiness*, poses significant challenges, particularly for those with limited exposure to pediatric care.^8^,^17^,^18^ Fear of working with children, insufficient preparation, and moral distress contribute to attrition rates among graduate nurses.^4^,^8^,^15^,^19^ High turnover rates among newly hired registered nurses underscore the importance of addressing these issues, as nurses exposed to moral distress are more likely to leave the profession.^20^ Adequate preparation for the role of pediatric nursing is crucial to mitigate moral distress and improve retention rates among new graduate pediatric nurses.^21^

There have been recent calls for action to support the recruitment and retention of a qualified pediatric nursing workforce through education.^14^ A preliminary search of MEDLINE, the Cochrane Database of Systematic Reviews, and *JBI Evidence Synthesis* was conducted and no current or in-progress systematic reviews or scoping reviews on this topic were identified. To address that gap, this scoping review aimed to identify and map the breadth of evidence available on the preparation of preregistration nursing students to provide care to infants, children, young people, and their families in an Australian context.

## Review questions

- What are the regulatory requirements, and professional and industry recommendations, for preparing preregistration nursing students to care for infants, children, young people, and their families in the clinical setting?
- How are preregistration nursing students prepared to care for infants, children, young people, and their families in a clinical setting?
- What are the perceptions and experiences of preregistration nursing students on clinical placements in areas where infants, children, young people, and their families receive care?

## Eligibility criteria

### Participants

This scoping review considered published and unpublished literature involving UG preregistration nursing students, including those enrolled in dual nursing/midwifery preregistration courses. Government and industry sources where the workforce was defined as all members of the multidisciplinary health care team, including students, were considered. For example, the Australian Commission on Safety and Quality in Health Care (ACSQHC) national standards outline workforce requirements to care for infants, children, and young people, and the workforce definition included students.^1^

### Concept

This scoping review considered literature relevant to the preparation of UG students to provide nursing care for infants, children, young people, and their families. For this review, the team defined *preparation* as curricula and clinical, simulation-based, policy-mandated, or practice-based activities intended to develop students’ knowledge, skills, confidence, communication, and capability to care for infants, children, young people, and their families. This definition was developed by review team consensus using our experience in pediatric nursing and nursing education. *Nursing care* was defined as care provided within the nursing students’ scope of practice, and according to the Nursing and Midwifery Board of Australia Registered Nurse Standards for Practice.^22^ Infants were defined as newborn to <1 year, children as 1 to <13 years, and young people as 13 to 16 years, which is generally the age limit for pediatric health services in Australia.^23^ Family-related content included family-centered care, communication with parents/caregivers, partnership in care, family assessment, education, support, advocacy, and shared decision-making.

The review also considered regulatory requirements, professional recommendations, and industry recommendations relevant to such preparation, as well as nursing students’ perceptions and experiences of preparation and clinical placement.

### Context

This scoping review considered literature reporting any setting where an infant, child, or young person may receive nursing care, including primary, secondary, or tertiary health care settings and community and mental health services. This scoping review was limited to the Australian context due to the comprehensive preparation of preregistration nursing students, in the absence of specialist nursing registration pathways, to enable workforce flexibility.^24^

### Types of sources

This scoping review considered all evidence sources relevant to the eligibility criteria: empirical research (studies with systematic data collection and analysis), non-empirical literature (conceptual, theoretical, or opinion-based works), policy or practice documents (such as guidelines, frameworks, and standards), and gray literature (materials published outside traditional academic channels). Sources were identified through electronic database and advanced Google searches, as well as searches of professional organizations, pediatric hospitals, and government institutions representing the care of infants, children, and young people in Australia.

## Methods

This review was conducted in accordance with the JBI methodology for scoping reviews,^25^ and is reported in line with the Preferred Reporting Items for Systematic Reviews and Meta-Analyses extension for Scoping Reviews (PRISMA-ScR).^26^ A scoping review was chosen to assess and comprehend the scope of knowledge as there is limited published research on this topic, and because a scoping review can encompass various types of literature beyond just research studies.^27^ This review was conducted in accordance with an a priori protocol (https://osf.io/4mtf6/files/wq5k4). A deviation from the a priori protocol occurred as quality appraisal was not performed, primarily because such an appraisal is contrary to the JBI scoping review methods and because it is unnecessary to include in order to facilitate readers’ understanding of the findings.

### Search strategy

The search strategy aimed to locate both published and unpublished literature.^25^ An initial limited search of MEDLINE (Ovid) and CINAHL (EBSCOhost) was undertaken to identify relevant peer-reviewed Australian articles on the topic, of which 2 were found.^4^,^19^ The search strategy was guided by the following 5 concepts: nursing students, clinical placement, children, preparation, and Australia. The text words contained in the titles and abstracts of relevant articles, and the index terms used to describe the articles, were used to develop a full search strategy under the guidance of a Faculty of Health Science librarian (VV). The search strategy, including all the identified keywords and index terms, was adapted for each information source, and a second search was undertaken to determine if the results from each selected information source included the 2 originally identified studies. The full search strategies are provided in Appendix I. The reference lists of sources included in the review were screened for additional studies.

Sources published from 2010 to December 2023 were included, as this start date aligned with the creation of the Australia Nursing and Midwifery Accreditation Council (ANMAC), which defined national education and registration standards for nurses in Australia.^24^ The databases that were searched included CINAHL (EBSCOhost), MEDLINE (Ovid), Scopus, and ERIC (ProQuest). Sources of unpublished and gray literature searched included the websites of Australian professional organizations, government bodies, pediatric hospitals, and universities, along with advanced Google searches.

The search strategy for unpublished studies, the Australian Government, health care organizations, and professional organizations was conducted in January 2024 and followed an iterative approach, where findings from the initial search were used to refine search terms and identify additional sources, which involved collaboration among the reviewers to adapt the keywords to each source. This included a Trove search by reviewer YK in February 2024 to identify theses. Two reviewers (YK and HZ) conducted an advanced Google search and simultaneously screened the first 30 records. The websites of professional organizations relevant to nurses who care for infants, children, and young people were searched by reviewer MY using the keywords *nursing student, student, nurse*, and *workforce*/*staff*. Australian pediatric hospitals were identified by Google searches, and hospital websites were searched by MY using the keywords *nursing student placement, nursing student*, and *student*. Australian tertiary nursing schools were identified by Google searches, and their websites were searched by MY using the keywords *nursing, children*, and *paediatrics*. Relevant websites were captured via screenshot to ensure an accurate record of available course information at the time of the review. Data were then extracted from publicly available sources, such as course handbooks, unit titles, and course structures, to identify discrete and/or embedded pediatric content.

### Source of evidence selection

Following the search, all identified records were collated and uploaded into EndNote v21.2. (Clarivate Analytics, PA, USA). The citations were then transferred to Covidence (Veritas Health Innovation, Melbourne, Australia) and duplicates removed. Prior to the screening process, 3 reviewers (MY, YK, and HZ) undertook a pilot test, concurrently screening the titles and abstracts of the first 25 articles, sorted by author, to ensure consistency between the reviewers and to refine the screening criteria. Following the pilot test, titles and abstracts were screened by 2 reviewers (MY and YK) independently for assessment against the eligibility criteria for the review. Potentially relevant sources were retrieved in full and their citation details imported into Covidence. The full-text of the selected citations was assessed in detail against the eligibility criteria independently by the same 2 reviewers (MY and YK). Sources of evidence that did not meet the eligibility criteria were excluded, and reasons for their exclusion are provided in the PRISMA flowchart and Appendix II.^28^ Any disagreements that arose between the reviewers were resolved through discussion or with a third reviewer (HZ), with confirmation from a fourth reviewer (JB). Hand-searching of the reference lists from the included articles was performed using the same screening process. Gray literature was reviewed with discussion between the review team to establish the relevance and quality of information.^29^

### Data extraction

The data extraction process was calibrated using the first 3 included sources. All 4 reviewers collaborated to extract data (see https://osf.io/4mtf6/files/wq5k4), with disagreements resolved through discussion until consensus was reached, after which the data extraction tool was refined. One reviewer (YK) then extracted data from the remaining sources using the finalized tool, with the extracted data verified by a second reviewer (MY, HZ). The data extracted included the type of evidence source and specific details about the participants, study settings, data collection methods, data analysis methods, outcome measures, and results. Key findings relevant to the scoping review concepts and questions were also extracted, such as the nature of the preparation described (eg, curriculum content, simulation, placement, mandatory training, alternative experiential learning) and nursing students’ perceptions and experiences of preparation or clinical placement. Data obtained from website searches, gray literature, and government and professional organizations were extracted into separate tables, including details about the source, information type/year, and content. The contents of the final data extraction tables were re-examined, cross-checked and refined by the review team, which strengthened the data abstraction and synthesis. Finally, the data were characterized using the 3 phases of content analysis described by Elo and Kyngäs in Peters *et al*.^25^: preparation, organizing, and reporting, based on the review questions.

### Data analysis and presentation

The extracted data were mapped to the 3 categories outlined in the priori protocol and aligned with the review questions: regulatory requirements and professional and industry recommendations; approaches used to prepare preregistration nursing students; and students’ perceptions and experiences of preparation and clinical placement.

## Results

### Source of evidence inclusion

A total of 39 sources were included in the review. These are broken down by database searches for peer-reviewed articles, citation searching of included articles, searches of theses and advanced Google searches, searches of government and professional organization websites, searches of Australian pediatric hospital websites, and searches of Australian university school of nursing websites.

#### Peer-reviewed articles

A total of 193 articles were identified via electronic database searches (Figure 1).^28^ Articles were uploaded to Covidence, and 51 duplicates were automatically removed. Eight duplicates were removed manually. The remaining 134 articles were screened for eligibility by title and abstract: 118 articles did not meet the eligibility criteria. Sixteen articles were retrieved and assessed for their eligibility. Eight articles were excluded for reasons of study population (n = 3), ineligible concept (n = 3), and ineligible context (n = 2). From the reference lists of the remaining 8 articles,^4^,^13^,^19^,^30^-^34^ we identified 2 further studies.^35^,^36^ The final 10 peer-reviewed articles included 5 qualitative scholarly studies,^13^,^30^,^32^-^34^ 3 mixed methods studies,^4^,^19^,^31^ an integrative review,^35^ and a systematic review.^36^ Characteristics of the peer-reviewed sources are provided in Appendix III.

**Figure 1:**
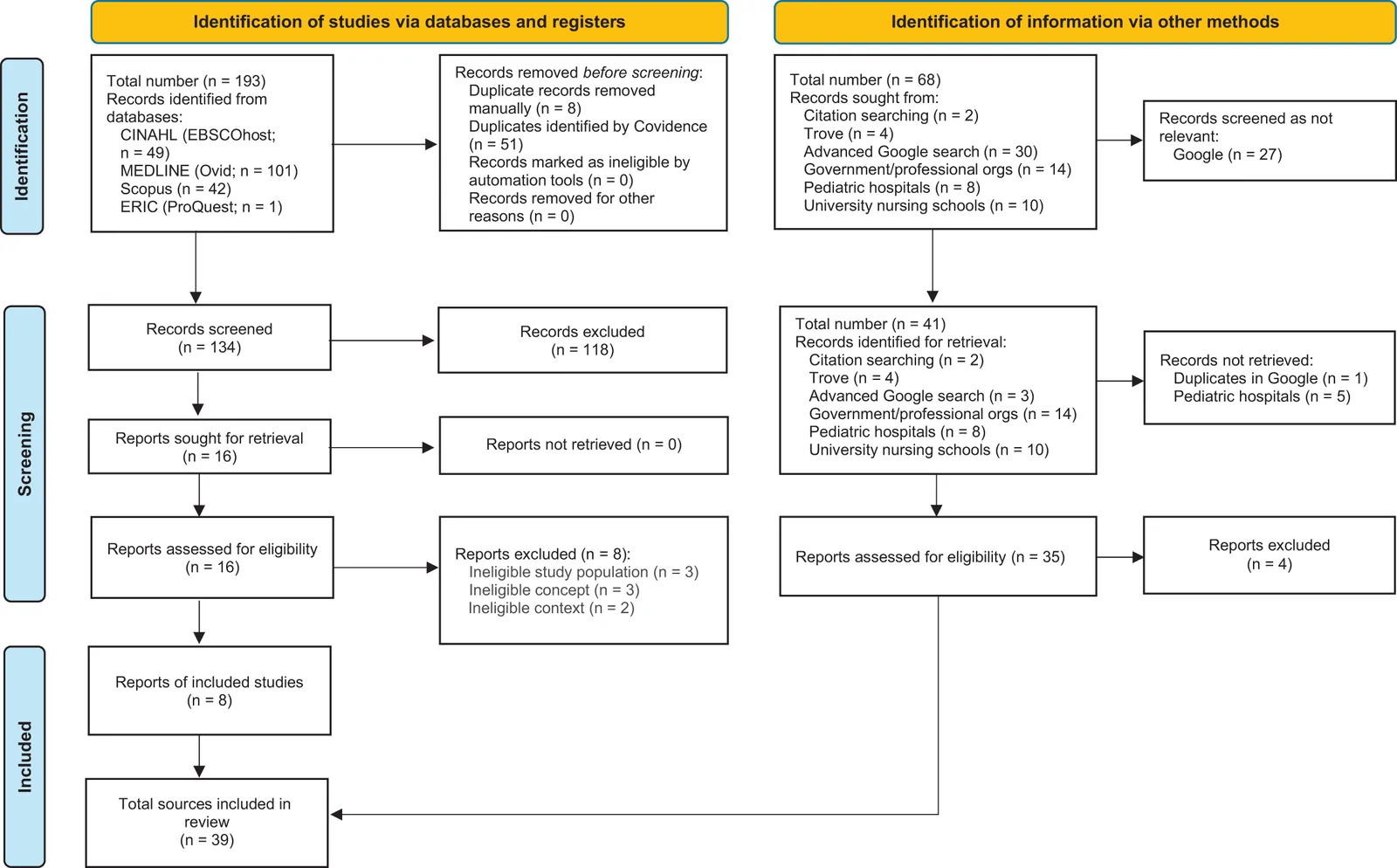
Search results and source selection and inclusion process^28^

#### Theses and advanced Google searches

The search identified 4 research theses through Trove, which were excluded because they did not meet the eligibility criteria for participants (n = 2), context (n = 1), and concept (n = 1). Advanced Google searches identified guidelines for nursing students about pediatric medication by the University of South Australia^37^ and an anonymous nursing student’s perceptions and experiences of pediatric placement according to a written reflection shared via a blog titled “The Footnotes.”^38^

#### Government and professional organizational websites

Four professional bodies representing nurses who care for infants, children, young people, and their families were identified by researcher collaboration, which formed the first stage of the search: the Australian College of Children’s and Young Peoples Nurses (ACCYPN), Australian Association for Infant Mental Health (AAIMH), ACSQHC, and ANMAC. Iterative searching^29^ identified the following additional sources: the First 2000 Days framework; the World Association for Infant Mental Health (WAIMH); the Australian Childhood Maltreatment Study; the Association for the Welfare of Children in Hospital (AWCH); and the New South Wales (NSW) Government Health, Education and Training Institute (HETI) matrices and guidelines. Fourteen records from the listed sources were retrieved and categorized into tables according to the scoping review questions.^1^,^24^,^39^-^50^ In summary, professional and government websites contained 3 policy documents,^45^-^47^ 1 accreditation standard^24^ and 2 practice standards,^42^,^49^ 2 conference abstracts,^39^,^40^ 2 position statements,^41^,^44^ a competency guideline,^43^ a user guide,^1^ a report,^48^ and mandatory training modules^50^ (see Table 1).

**Table 1:** Included evidence sources from database, Google Advanced, professional organizational and government, and Australian pediatric hospital website searches

Evidence source	Evidence type	Topic
Databases	Qualitative scholarly studies	Semi-structured interview to evaluate a breastfeeding learning module^30^
		Semi-structured interview to retrospectively evaluate student placement experience once they had been employed^13^
		Observation of student participation in a neonatal resuscitation and parental bereavement simulation^32^
		Interviews, meeting minutes, documentation, and researchers’ reflective journal during a practicum experience^33^
		Interviews, focus group, and open-ended questionnaires to review academic staff, nursing staff, and student experiences during and after a practicum experience^34^
	Mixed methods scholarly studies	Open-/closed-ended questionnaire to evaluate a student-led medical play activity with children^4^
		Immediate, post placement, and post-employment evaluation survey of a student-led simulated ward experience^19^
	Mixed methods study	Cross-sectional survey to evaluate student knowledge, attitudes, and beliefs about LGBT parents^31^
	Integrative review	Student knowledge of breastfeeding^35^
	Systematic review	Student knowledge of breastfeeding^36^
Google Advanced	Guideline	Medicine administration and scope of practice guidelines for students^37^
	Anonymous student reflection	Describes the experience of pediatric placement, discovering passion for pediatric nursing, and the value of compassion, empathy, building trust with patients and their families, and going above clinical responsibilities^38^
Professional organizational website: ANMAC	Accreditation standard	Registered nurse preparation aims to produce a mobile and generalist workforce and safety for the public without the need for specialist training to care for infants and children. To be accredited, the program’s content and subject learning outcomes ensure integrated knowledge of care across the lifespan and across contexts of nursing practice^24^
Professional organizational website: ACCYPN	Conference abstracts	International pediatric placement experiences offer immersion with continuous support from academic supervisors^39^
		Community pediatric placement experiences offer unique and valuable experiences with continuous support from academic supervisors^40^
	Position statement	Education and continuous professional development affect the quality of care delivered by nurses who provide care to children, young people, and their families across various settings. It is essential for nurses to be well-prepared for both general and specialized practice^41^
	Practice standard	Outlines the minimum requirement expected of all professionals working with children, young people, and their families to ensure their best possible health and psychological well-being^42^
Professional organizational website: AAIMH	Competency guidelines	Infant mental health^43^
Professional organizational website: WAIMH	Position statement	Rights of infants^44^
Professional organizational website: AWCH	Practice standard	Aims to recognize that the medical and psychosocial needs of children and adolescents differ from those of adults and that this is reflected in the health services’ provision of safe, quality care through appropriately trained staff^49^
Government website: NSQHS	User guide	Acute and community health service organizations that provide care for children^1^
Government website: NSW Health	Policy documents	The safety and wellbeing of children and adolescents in NSW acute health facilities^45^
		Matrix and targeting guide: outlines mandated learning for nurses working with children and their families, including nursing students attending practicum^46^
		NSW implementation framework for the first 2000 days relevant to nurses working with children and their families, including nursing students attending practicum^47^
	Training modules	Online training for mandatory requirements outlined in NSW policy document for nurses working with children and their families, including nursing students attending practicum^50^
Government website: NHMRC	Report	Australian Childhood Maltreatment Study: health practitioners require preservice training and ongoing education to understand, identify, and respond to child maltreatment^48^
Australian pediatric hospital websites	Policy documents	Students on clinical placement^51^
		Explicit policies and agreement for students, undergraduate clinical practice manual,^52^ and Undergraduate student scope of practice document^53^
		Commitment to supporting students for future workforce - Clinical teaching and learning strategy document^54^

AAIMH, Australian Association for Infant Mental Health; ACCYPN, Australian College of Children’s and Young Peoples Nurses; ANMAC, Australia Nursing and Midwifery Accreditation Council; AWCH, Association for the Welfare of Children in Hospital; LGBT, lesbian, gay, bisexual, and transgender; NHMRC, Australia’s National Health and Medical Research Council; NSQHS, Australia’s National Safety and Quality Health Service Standards; NSW, New South Wales; WAIMH, World Association for Infant Mental Health.

#### Australian pediatric hospital websites

Eight Australian pediatric hospitals were identified by Google searches: Queensland Children’s Hospital, Sydney Children’s Hospital, Royal Children’s Hospital Melbourne, Mater Children’s Private Brisbane, Centenary Hospital for Women and Children Canberra, Perth Children’s Hospital, Children’s Health Queensland Hospital and Health Service Brisbane, and Women’s and Children’s Hospital North Adelaide. Hospital websites were searched, and policy documents were identified and retrieved from 4 of those websites.^51^-^54^

#### Australian university school of nursing websites

Google searches for Australian tertiary nursing schools identified Curtin University, Deakin University, Edith Cowan University, Griffith University, Murdoch University, Notre Dame University, Queensland University of Technology, University of Melbourne, University of Queensland, and Western Sydney University as the first 10 Universities. Information was retrieved from the university websites (Table 2).^55^-^64^

**Table 2:** Publicly available nursing program information extracted from Australian university websites

University	Undergraduate	Elective	Postgraduate
Curtin University^55^	Integrated lifespan approach	—	—
Edith Cowan University^56^	Third year: Children’s and young people’s health and well-being	—	Graduate certificate in children’s and young people’s nursing
Notre Dame University^57^	—	Maternal care	—
		Pediatric nursing	
Murdoch University^58^	First year: Lifespan unit	—	—
	Second year: Maternity, child, and adolescent nursing care		
Western Sydney University^59^	—	Maternal and infant health care	—
Deakin University^60^	First year: Care of the child and adolescent	—	—
Griffith University^61^	First year: Lifespan unit	Third year elective:	Available to be studied as double degree
	Second year: Family nursing		
		Child and youth mental health Care of the neonate	
Queensland University of Technology^62^	First year: Wellness across the lifespan	Specialist pediatric and adolescent nursing	—
University of Queensland^63^	First year: Lifespan unit	—	—
	Third year: Lifespan unit		
University of Melbourne^64^	—	Specialist certificate in pediatric nursing	Graduate certificate in nursing practice—pediatric

### Review findings

Data extraction matrices for answering the review questions are provided separately for peer-reviewed sources (Appendix IV), theses and Advanced Google searches (Appendix V), government and professional organization websites (Appendix VI), and Australian pediatric hospital websites (Appendix VII).

#### Regulatory requirements, professional recommendations, and industry recommendations

##### Scholarly literature

Recommendations from the scholarly articles in this review included providing education regarding breastfeeding^30^,^35^,^36^ and using educational strategies to increase awareness and understanding of family diversity.^30^ Further recommendations included the use of simulated learning to develop nursing students’ confidence in preparation for placement,^4^,^19^,^31^ work-integrated learning through supported clinical placements,^30^ and primary care placements delivered between the university and industry.^32^,^33^

##### Government and professional organizations

ANMAC requires that tertiary nursing program content and subject learning outcomes ensure an integrated knowledge of care across the lifespan and across contexts of nursing practice. The aim of this requirement is to produce a mobile and generalist workforce and ensure safety for the public without the need for specialist training to care for infants and children.^24^ The ACSQHC includes nurses and students within the definition of the workforce, and recommends the necessary skills, knowledge, experience, and clinical guidelines for safe child and family-focused health care, which must be supported by governance, including the provision of pediatric guidelines.^1^

While recommendations for health care practitioners, including student nurses, were found on the websites of the AWCH, AAIMH, and WAIMH, the ACCYPN, which represents nurses who care for infants, children, and their families in Australia, was the only professional organization that made recommendations specifically for the UG preparation of nurses. The ACCYPN Nurse Education Position Statement^41^ states the following:

Nurses play a significant role in the delivery of health care to children, young people and their families in a variety of clinical settings. Nurse education and ongoing professional learning impacts on the quality of health care provided for these client/patient groups. There is an ongoing need for nurses to be appropriately prepared for generalist and specialty practice.^(p.1)^

As such, curriculum design and assessment are recommended to enable graduates to meet the ACCYPN’s minimum practice standards for nurses caring for children.^42^

Common features in the recommendations on the websites of government and professional organizations include an understanding of the physical, psychosocial, and developmental needs of children and young people^1^,^42^,^44^,^45^,^47^-^49^ and an awareness of the risks to development.^42^,^44^ The importance of learning developmentally appropriate communication skills was highlighted, as well as the importance of the multidisciplinary team working in partnership with families to provide child- and family-centered care.^1^,^42^,^44^,^45^,^47^-^49^ Knowledge regarding advocacy for the rights of the child,^42^ culturally sensitive care,^45^ and legal and ethical obligations, including consent, was also recommended.^1^

Concerning safety, the clearance to work with children was mandated or recommended across a number of government sites and professional organizations, as was education regarding environmental safety, harm minimization, recognition and response to deterioration, pediatric basic life support, medication calculations and safe administration, infection prevention and antimicrobial stewardship, and child protection, including the recognition of child abuse and mandatory reporting.^1^,^37^,^42^,^46^,^49^

Professional organizations also recommended educational preparation with reference to mental health and social concerns,^1^,^43^,^44^,^48^ specifically mental health assessment, trauma-informed care, end-of-life care,^1^ and infant mental health.^43^,^44^ Promoting professional well-being through supervision and reflection was advocated by infant mental health organizations,^43^,^44^ as well as access to clinical expertise^1^,^39^-^42^,^45^,^46^,^49^ and appropriate preparation of clinical facilitators.^42^-^46^

##### Pediatric hospitals

Industry recommendations from pediatric hospitals included the clearance to work with children, basic life support, and mandatory reporting requirements,^51^-^53^ as well as recognition of deterioration, child protection, and medication safety.^51^

#### Preparation approaches for preregistration nursing students

##### Scholarly literature

The interventions reported to prepare nursing students to care for infants, children, young people, and their families can be categorized into education, simulation, and work-integrated learning. The literature described innovatively designed breastfeeding education through a range of formats and educational strategies that link knowledge to practice, based on the World Health Organization’s 10 Steps to Successful Breastfeeding,^30^,^35^,^36^ as well as educational interventions that prepared students to care for children of lesbian, gay, bisexual, and transgender parents with open-minded, nonjudgmental, and respectful attitudes.^31^

Preparation approaches included simulation-based learning, such as neonatal resuscitation with family communication and support during a bereavement situation,^32^ engaging with children in a play-based ward simulation,^4^ and participating in care in which nursing students made decisions on how to maintain a safe environment.^4^,^19^ Clinical placement alternatives in which nursing students worked in collaboration with academics or nursing specialists to provide care were described. Interventions included a camp for children with chronic conditions^34^ and a community engagement placement with a family who had a child with a disability.^33^

##### Government and professional organizations

Further preparation approaches, according to organizational contributions, are an international rural primary care–based experience where nursing students worked with academics on health promotional activities and child assessments,^39^ and a community placement where nursing students interacted with children who had chronic kidney disease and their families, observing the multidisciplinary care team, complexities of care, and impact of chronic illness.^40^ Commonly, these experiences provided nursing students with the opportunity to develop interactive skills to prepare them to work with children and families while being supported by an experienced academic.^39^,^40^

The NSW Government website^45^ provides a policy document stating that children and adolescents must be cared for by staff with appropriate skills and requires core training in pediatric resuscitation, recognition of deterioration, child protection, and medication safety. Accompanying training modules are provided by the NSW Government HETI to all staff, including nursing students, prior to caring for infants, children, young people, and their families.^45^-^47^,^50^

##### Pediatric hospitals

Three Australian pediatric hospitals provided policy documents, scope of practice information, and guidelines for nursing students on clinical placement.^51^-^53^ Sydney Children’s Hospital was the only hospital to provide publicly available details of the preparation of nursing students: the core training modules mandated by the NSW Government completed during nursing student orientation, eLearning prior to nursing student placement, and preparation of clinical facilitators to adequately support nursing students while on placement.^51^ Three other hospitals had publicly available policy documents outlining the scope and support of nursing students in pediatric areas, although we could not identify specific details about the preparation of nursing students prior to placement.^52^-^54^

##### University schools of nursing

University websites showed that nursing students are prepared to care for children and their families with an integrated lifespan approach, which is taught throughout the curriculum,^55^ via a discrete lifespan unit^58^,^61^-^63^ or via a discrete pediatric unit.^56^,^58^,^60^ In addition, some universities provide an elective pediatric unit/program to nursing students who self-select that option.^57^,^59^,^61^,^62^,^64^

#### Students’ perceptions and experiences

##### Scholarly literature

Nursing students perceived limitations in their knowledge of breastfeeding, which affected their confidence and ability to support families, and described feeling more positive following breastfeeding curriculum interventions.^30^,^35^,^36^ Moreover, nursing students felt they knew less than other health professions students about working with families, including parents and caregivers of diverse genders and sexualities.^31^

Nursing students perceived that simulation experiences enhanced their communication,^4^,^32^ confidence,^4^,^32^ preparation and readiness for clinical work,^4^,^19^ clinical reasoning and skills,^19^ and feelings about working in this area.^4^,^32^ Simulations^4^,^19^,^32^ and work-integrated learning activities^33^,^34^ were also identified by students as enabling self-discovery of their attitudes and reactions to different situations.

Clinical support and facilitation while on placement, combined with opportunities for education and debriefing, assisted nursing students in linking theory to practice,^30^ increased their appreciation for the child’s perspective, and supported their understanding of how to work with a family.^33^ Students felt that positive clinical experiences also influenced their choice of whether to work in pediatrics in the future.^13^,^32^,^33^

##### Government and professional organizations

According to organizational contributions, students described experiencing improved confidence and empathy after working with pediatric patients in alternative clinical placements.^39^,^40^

##### Individual experiences

A personal student reflection described the impact of a positive clinical placement experience, which extended beyond clinical responsibilities to fostering compassion, empathy, and trust-building with patients and families.^38^

## Discussion

This scoping review aimed to record the extent of the literature and other sources of information regarding the preparation of preregistration nursing students to provide care to infants, children, young people, and their families in an Australian context. A limitation of this scoping review is that it relied on publicly available information from hospitals, government organizations, and universities, which may not fully reflect the actual preparation and support provided to nursing students. There were more sources identified regarding the recommendations for nursing students than the actual preparation of nursing students, and there were limited scholarly articles regarding the preparation of student nurses to care for children and their families overall. Very little has been published regarding the experiences of nursing students caring for children on placement in Australian institutions. Internal resources, informal support systems, and institution-specific practices may not be accessible or published, potentially limiting the completeness and accuracy of the findings. Figure 2 illustrates the nature and quantity of the sources included in this review.

**Figure 2:**
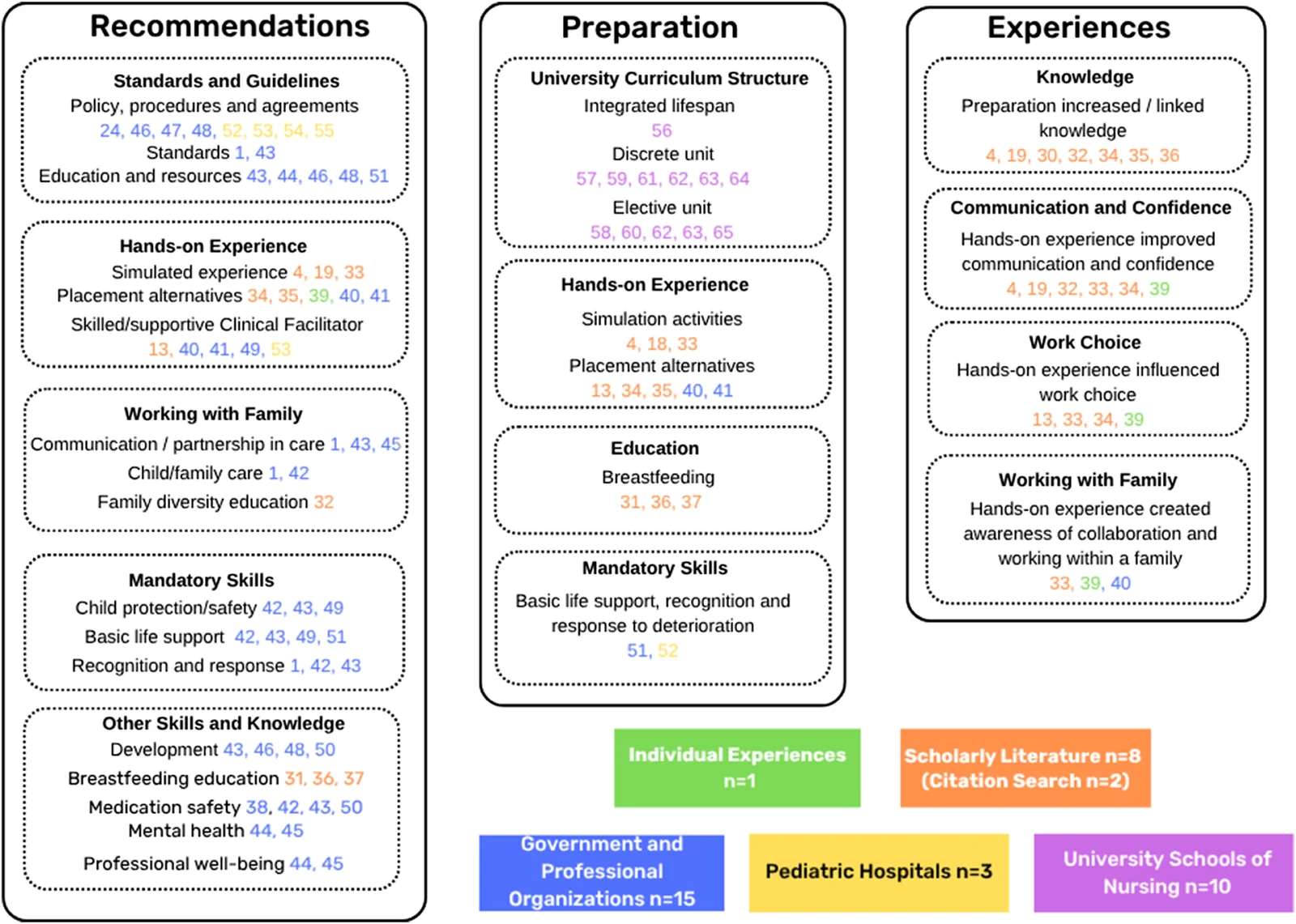
Integration of the recommendations, preparation, and experiences of undergraduate nursing students regarding the care of infants, children, young people, and their families. Reference numbers are color-coded by source category.

### Requirements and recommendations for the preparation of nursing students

The requirement for graduating nurses in Australia to have an integrated knowledge of care across the lifespan^22^ relies on specialist education at the postgraduate level to develop pediatric nursing skills. Through such specialist education, student nurses may also build an understanding of the vulnerability of children and young people and the careful handling required as generalists rather than experts.^65^ There are currently no specific requirements for registered nurses to be able to care for infants, children, and their families on graduation in Australia,^66^ and the need for support to develop nurses’ capability in this regard arose as a recurring theme in the 2019 review of health services for children, young people, and families within the NSW Health system.^67^

Most of the recommendations and requirements made by the industry, government, and professional organizations, as captured in this scoping review, are related to the safety and protection of children, including mandatory training in child protection, pediatric basic life support, and medication safety.^1^,^37^,^42^,^45^,^47^-^49^ However, an integrative review by Lines *et al*.^68^ identified that training alone is not the best way to prepare nurses for their role in safeguarding children; beyond that, ongoing clinical education and organizational support are required to build their confidence and ability to advocate for children and respond to safety concerns. An understanding of development was recommended in policy documents and competency guidelines, along with communication and collaboration skills enabling health professionals to work in partnership with a child and their family.^1^,^42^,^43^,^45^ These have been identified in the wider literature as the cornerstones of accomplishing safe pediatric care.^69^

Increased nursing student contact and exposure to children and families while being supported by an experienced clinical facilitator or an academic was recommended^42^ and described as beneficial to include in simulated learning^4^,^19^,^32^ and supported work-integrated learning experiences.^33^,^34^,^39^,^40^ With limitations on the availability of pediatric clinical placements,^4^ immersive experiential learning opportunities may offer an alternative to prepare nursing students while addressing the equity of access for students from rural, remote, or other marginalized populations. In a systematic review of nursing students’ evaluations of clinical placements,^70^ authentic learning was found to be more important for the support and supervision of students than the availability of clinical nursing tasks. Furthermore, high-fidelity simulation helped nursing students increase their knowledge, enhance their professional skills, and cultivate critical thinking, communication skills, and clinical judgment.^70^

### Preparation of nursing students

According to the findings of this scoping review, the evidence available on Australian university websites regarding the preparation of UG nursing students to care for infants, children, young people, and their families is inconsistent, with pediatric content either integrated or within a discrete lifespan unit. The former option, an integrated curriculum, follows constructivist learning theory. Scurlock-Evans and colleagues^71^ found no significant differences in evidence-based practice when comparing integrated and discrete curriculum designs. However, an integrated curriculum can be sensitive to the availability and timing of clinical placements. With that said, the integrated approach has been highlighted as a cause of the decline in pediatric and child health content in the UG curriculum and loss of staff with the experience and expertise needed to competently educate nursing students about the comprehensive care needs of children, adolescents, and their families.^15^

Alternatively, offering pediatrics as an elective unit provides education to a select group of nursing students who opt in, potentially leaving other nursing students to graduate without having had the opportunity to develop the generalist skills and knowledge required to recognize the vulnerability of children and their families and the care differences required for these groups.^65^ Interestingly, only 3 of the 10 university websites in this scoping review were found to offer postgraduate education in pediatric nursing,^56^,^61^,^64^ which may also contribute to the difficulty in employing academic staff to provide pediatric nursing education at the UG level.^15^

This scoping review identified simulation and alternative clinical placement experiences in which nursing students are immersed with children and families, which assists them in gaining confidence and developing communication and interpersonal skills.^4^,^19^,^32^-^34^,^39^,^40^ Simulation is known to be valuable when educators have the knowledge, skills, expertise, and technological support to incorporate high-quality simulation experiences throughout nursing programs.^72^ Similarly, alternative placements with adequate educational support can provide nursing students with the time and confidence to develop communication skills and build a rapport, and to develop confidence in working with children and families.^73^,^74^ Improved confidence was linked to positive feelings about working in this area, and hence, it is essential for the future pediatric nursing workforce,^4^,^13^,^32^ particularly for those without previous experience with children. On this topic, Al Touby found that an alternative pediatric clinical placement experience increased male students’ skills and knowledge and fostered their confidence in pursuing future careers as pediatric nurses.^75^

The significance of the clinical facilitator/teacher role has been clearly reported in the literature historically.^76^ It was identified in this review as an important factor supporting nursing students in developing the skills and confidence to care for children, which they developed by working in collaboration with academics and experienced nurses.^33^,^34^ Additionally, supported work-integrated learning experiences were shown to increase students’ appreciation for the child’s perspective and their understanding of nursing within a family.^33^ An academic-clinical partnership model has been implemented with some success in maternal-newborn nursing, which is reported to address UG curriculum limitations and workforce issues by supporting nursing students, while also improving education, nurturing the professional growth of experienced nurses, and promoting safe, high-quality care.^77^ Such collaborative models have been recommended in a recent report on the Australian professional workforce.^65^

Despite the recommendations and requirements, pediatric hospital websites demonstrate a potential lack of preparation of nursing students to care for children and their families prior to clinical placements in Australian hospitals. The provision of mandatory training and eLearning was identified on only 1 of the hospital’s websites,^51^ in alignment with policies and agreements from that state government’s health department that all students attending placements in pediatric areas receive training in pediatric basic life support, recognition and response to deterioration, and mandatory reporting.^46^,^47^,^50^ It can be assumed that the responsibility for completing mandatory requirements in many cases falls to the tertiary education sector or to the individual nursing student.

### Experiences of undergraduate nursing students

There was an absence of information on the experiences of nursing students regarding their preparation to provide care in a pediatric setting. Aside from an anonymous student reflection, reports of nursing student experiences were mostly from studies in which an intervention was being evaluated. Nonetheless, nursing students reported experiencing knowledge-linking,^4^,^32^,^34^,^35^ improved communication and confidence,^4^,^19^,^32^-^34^ positive influences on their work choice,^13^,^32^,^33^,^38^ and a developing awareness of working within a family^33^,^39^,^40^ through participation in simulation and supported work-integrated learning opportunities.

The common elements in positively evaluated simulation and supported work-integrated learning interventions were time, exposure, and experience with children and families, while being supported to develop and implement knowledge and skills by an academic or experienced nurse.^13^,^32^,^33^,^38^ This aligns with existing evidence on the value of experiential learning through simulation to develop nursing students’ skills and knowledge, although there is limited evidence regarding the retention of that knowledge.^78^ However, the positive experience may assist nursing students in identifying pediatric nursing as a potential career choice and may improve their experience of caring for children and their families in the absence of clinical placement experience and with a reduction in pediatric curriculum content.^15^ In particular, these interventions may assist nursing students in overcoming anxiety and challenges, lower moral distress and attrition rates among new graduates, and address professional burnout and workforce issues, which are clearly documented in the pediatric nursing literature.^4^,^8^,^10^,^11^,^13^

## Conclusions

The clearly defined regulatory requirements and recommendations from government and professional organizations state that health professionals in Australia should be trained in the safety and protection of people under their care across the whole lifespan. Yet there is limited information about the preparation of nursing students in Australia to care for children and their families, and limited studies report the perceptions and experiences of nursing students on pediatric placements. This scoping review identified the critical components of the training that these students require, which include safety, communication, working with families, and clinical experience or alternatives that are supported by an academic or expert nurse. To prepare nursing students to care for children and their families, educational institutions should integrate pediatric education into the UG curriculum with mechanisms for students to obtain experience in the absence of clinical placements.

### Implications for research

The paucity of the literature included in this review, with regard to both its depth and its breadth, warrants further primary studies, such as longitudinal studies tracking career pathways, intervention studies testing experiential learning, and qualitative studies of Australian nursing students’ experiences on pediatric clinical placements.

### Implications for practice

Findings from this review could inform the enhancement of ANMAC accreditation requirements, which may require more comprehensive Australian UG nursing education across the lifespan, including experiential learning hours (in the form of simulation, clinical placement, and alternatives) supported by experienced nurses and academic partnerships.

## Acknowledgments

Vanessa Varis, librarian of the Faculty of Health Science, Curtin University, for her assistance with the literature search.

## Funding

This scoping review was supported by a Curtin University Faculty of Health Sciences summer scholarship for a student research assistant (YESK). The role provided skill development in scoping review methods. No funding was provided for the included sources of evidence. Curtin University had no influence on the review and receives no financial benefit from its outcomes.

## Declarations

All reviewers hold current registration as registered nurses. Two reviewers have extensive experience in a variety of research methodologies, and 2 reviewers have extensive pediatric experience in wide range of clinical environments.

## Author contributions

MJY: concept, search strategy, screening, data extraction, data analysis, manuscript development; HZ: concept, search strategy, screening, data extraction, data analysis, manuscript review, project supervision; YESK: search strategy, screening, data extraction, data analysis, manuscript development; JAB: concept, screening, manuscript review, project supervision.

## Appendix I: Search strategy

**Table:**

CINAHL (EBSCOhost)		
Search conducted: December 24, 2023		
Search	Query	Records retrieved
#1	nurs* N3 (student* or undergraduat* or preregistrat* or “pre-registrat*” or bachelor* or prelicens* or “pre-licens*” or baccalaureate*)	37,460
#2	Clinic* N3 (practic* or placement* or fieldwork* or “work* learning” or “work* integrated learning” or internship* or setting*)	115,032
#3	paediatric* or pediatric* or child* or infant* or baby or babies or toddler* or adolescen* or teen* or youth* or “young people*” or “young person*”	979,476
#4	prepar* or readiness or skill* or knowledge or confiden* or competen* or simulat* or experienc* or tutorial* or workshop* or educat*	1,406,865
#5	australia* OR canberra OR “new south wales” OR sydney OR victoria OR melbourne OR queensland OR brisbane OR adelaide OR tasmania OR hobart OR perth OR darwin OR “northern territory”	133,480
#6	#1 AND #2 AND #3 AND #4 AND #5	49
Limited to a search range from 2010 to the search date, along with English-language limits

**Table:**

MEDLINE (Ovid)		
Search conducted: December 24, 2023		
Search	Query	Records retrieved
#1	nurs* ADJ3 (student* or undergraduat* or preregistrat* or “pre-registrat*” or bachelor* or prelicens* or “pre-licens*” or baccalaureate*)	64,446
#2	Clinic* ADJ3 (practic* or placement* or fieldwork* or “work* learning” or “work* integrated learning” or internship* or setting*)	644,007
#3	paediatric* or pediatric* or child* or infant* or baby or babies or toddler* or adolescen* or teen* or youth* or “young people*” or “young person*”	5,792,314
#4	prepar* or readiness or skill* or knowledge or confiden* or competen* or simulat* or experienc* or tutorial* or workshop* or educat*	7,681,337
#5	australia* OR canberra OR “new south wales” OR sydney OR victoria OR melbourne OR queensland OR brisbane OR adelaide OR tasmania OR hobart OR perth OR darwin OR “northern territory”	1,413,386
#6	#1 AND #2 AND #3 AND #4 AND #5	101
Limited to a search range from 2010 to the search date, along with English-language limits

**Table:**

Scopus		
Search conducted: December 24, 2023		
Search	Query	Records retrieved
#1	nurs* W/3 (student* or undergraduat* or preregistrat* or “pre-registrat*” or bachelor* or prelicens* or “pre-licens*” or baccalaureate*) AND	3086
#2	Clinic* W/3 (practic* or placement* or fieldwork* or “work* learning” or “work* integrated learning” or internship* or setting*) AND	1098
#3	paediatric* or pediatric* or child* or infant* or baby or babies or toddler* or adolescen* or teen* or youth* or “young people*” or “young person*” AND	60
#4	prepar* or readiness or skill* or knowledge or confiden* or competen* or simulat* or experienc* or tutorial* or workshop* or educat* AND	60
#5	australia* OR canberra OR “new south wales” OR sydney OR victoria OR melbourne OR queensland OR brisbane OR adelaide OR tasmania OR hobart OR perth OR darwin OR “northern territory”	42
Limited to a search range from 2010 to the search date, along with English-language limits. Searched W/3 within the article title, abstract, and keywords. Limit to Nursing.

**Table:**

ERIC (ProQuest)		
Search conducted: December 24, 2023		
Search	Query	Records retrieved
#1	nurs* NEAR/3 (student* or undergraduat* or preregistrat* or “pre-registrat*” or bachelor* or prelicens* or “pre-licens*” or baccalaureate*) AND	4128
#2	Clinic* NEAR/3 (practic* or placement* or fieldwork* or “work* learning” or “work* integrated learning” or internship* or setting*) AND	181
#3	paediatric* or pediatric* or child* or infant* or baby or babies or toddler* or adolescen* or teen* or youth* or “young people*” or “young person*” AND	10
#4	prepar* or readiness or skill* or knowledge or confiden* or competen* or simulat* or experienc* or tutorial* or workshop* or educat* AND	10
#5	australia* OR canberra OR “new south wales” OR sydney OR victoria OR melbourne OR queensland OR brisbane OR adelaide OR tasmania OR hobart OR perth OR darwin OR “northern territory”	1
Limited to a search range from 2010 to the search date, along with English-language limits.

**Table:**

Trove		
Search conducted: February 14, 2024		
Search	Query	Records retrieved
#1	(nurs* ~ (student* or undergraduate* or preregistrat* or “pre-registrat*” or bachelor* or prelicens* or “pre-licens*” or baccalaureate*))	0
#2	(Clinic* ~ (practic* or placement* or fieldwork* or “work* learning” or “work* integrated learning” or internship* or setting*))	**2**
#3	(pediatric* or paediatric* or child* or infant* or baby or babies or toddler* or adolescen* or teen* or youth* or “young people*” or “young person*”)	2
#4	(prepar* or readiness or skill* or knowledge or confiden* or competen* or simulat* or experience* or tutorial* or workshop* or educat*)	158
#5	#1 AND #2 AND #3 AND #4	4
Limited to a search range from 2010 to the search date, along with English-language limits.

**Table:**

Advanced Google search		
Search conducted: January 12, 2024		
Search	Query	Records retrieved
#1	(nursing student)(paediatric practicum|placement)(preparation)(infant|paediatric|child)	30

**Table:**

Professional organizational websites	
Search conducted: January 27, 2024	
Search terms: *nursing student*, then *student, nurse*, and *workforce*/*staff*	
Organization	Records
Australian College of Children’s and Young Peoples Nurses (ACCYPN)	4
Australian Association for Infant Mental Health (AAIMH)	1
World Association for Infant Mental Health (WAIMH)	1
Australian Commission for Safety and Quality in Health Care (ACSQHC)	1
Safety and Wellbeing of Children and Adolescents in New South Wales Acute Health Facilities	4
Australian Childhood Maltreatment Study	1
Australian Nursing and Midwifery Accreditation Council (ANMAC)	1
Association for the Welfare of Children in Hospitals (AWCH)	1

**Table:**

Australian pediatric hospital websites	
Search conducted: January 29, 2024	
Search terms: *nursing student placement, nursing student*, and *student*	
Hospital	Records
Children’s Hospital New South Wales	2
Children’s Hospital Victoria	2
Children’s Hospital Queensland	1
Children’s Hospital Australian Capital Territory	1
Children’s Hospital Western Australia	1
Children’s Hospital South Australia	1
Children’s Hospital Tasmania	0
Children’s Hospital Northern Territory	0

**Table:**

Australian university schools of nursing	
Search conducted: February 2, 2024	
Search terms: *Australian university nursing - Domestic Undergraduate* (first 10 universities)	
University	Records
Edith Cowan University WA	1
Notre Dame WA	1
Murdoch University WA	1
Western Sydney University	1
Deakin University	1
Curtin University	1
Griffith University	1
Queensland University of Technology	1
University of Queensland	1
University of Melbourne	1

## Appendix II: Studies ineligible following full-text review

### Peer-reviewed articles (n = 8)

1. Sheeran L, Ridgway L, Buchanan K, Wilson D. Consultation to review clinical placement processes: a quality assurance project. Nurse Educ Pract 2022;61:103331.
  *Reason for exclusion:* Ineligible study population.
2. Raines DA. A dedicated education unit for maternal-newborn nursing clinical education. Nurs Womens Health 2016;20(1):22-7.
  *Reason for exclusion:* Ineligible study population.
3. Tolchard B, Stuhlmiller CM. Outcomes of an Australian nursing student-led school vision and hearing screening programme. Child Care Pract 2018;24(1):43-52.
  *Reason for exclusion:* Ineligible study population.
4. Hutchinson M, Doran F. Psychometric testing of inventory of beliefs and attitudes on domestic violence. Nurse Res 2017;25(1):30-6.
  *Reason for exclusion:* Ineligible concept.
5. Lines LE, Bell A, Hunter S, Matwiejczyk L, Williams J, Kakyo TA, *et al*. Interprofessional education within a nurse practitioner led paediatric service: a multi-methods study. Nurse Educ Pract 2024;75:103909.
  *Reason for exclusion:* Ineligible concept.
6. Swift L, Henderson A, Wu CJJ. Self-confidence in clinical skill: a descriptive study of the perspective of first-year nursing students. Nurse Educ Pract 2022;58:103270.
  *Reason for exclusion:* Ineligible concept.
7. Happell B, Platania-Phung C. Mental health placements in a general health setting: no substitute for the real thing! J Clin Nurs 2012;21(13-14):2026-33.
  *Reason for exclusion:* Ineligible context.
8. MacKinnon K, Marcellus L, Rivers J, Gordon C, Ryan M, Butcher D. Student and educator experiences of maternal-child simulation-based learning: a systematic review of qualitative evidence. JBI Database Syst Rev Implement Rep 2017;15(11):2666-706.
  *Reason for exclusion:* Ineligible context.

### Trove search (n = 4)

1. Alexander FJ. An investigative study into the Advanced Certificate in Nursing (Enrolled Nurse) Course [MA thesis]. Macquarie University; 1999.
  *Reason for exclusion:* Ineligible study population.
2. Whiteing N, Barr J, Rossi DM. The practice of rural and remote nurses in Australia: a case study. J Clin Nurs 2022;31(11-12):1502-18.
  *Reason for exclusion:* Ineligible study population.
3. Sastrawan. Developing and evolving a value system: a grounded theory [PhD thesis]. Monash University; 2020.
  *Reason for exclusion:* Ineligible study population.
4. Valcan DS, Malpique A, Pino-Pasternak D, Asil M, Teo T. The contributions of executive functioning to handwritten and keyboarded compositions in Year 2 children. Contemp Educ Psychol 2024;77:102272.
  *Reason for exclusion:* Ineligible study population.

## Appendix III: Characteristics of included peer-reviewed sources

**Table:**

Evidence source	Study design	Data collection methods	Study setting	Participants (characteristics)	Intervention	Data analysis method	Outcome measures
Bowdler *et al*. 2023^30^	Qualitative: single case study, phenomenological design	Semi-structured interview to evaluate breastfeeding learning module	New South Wales tertiary institution	Final-year undergraduate nursing students enrolled in an elective pediatric unit (N = 9)	7-week online breastfeeding module	Thematic analysis—NVivo 12 (Lumivero, Denver, Colorado) software	Participant experience of the module content:
					Semantic wave module design to link students’ theory to practice		i) Threads and links
					Content based on 10 Steps to Successful Breastfeeding and the Australian Infant Feeding Guidelines.		ii) Engaging structure
							iii) Seedlings.
Bowdler *et al*. 2022^35^	Integrative review: student knowledge of breastfeeding	Databases: CINAHL, Scopus, Web of Science, MEDLINE, PubMed, and an advanced library search (all EBSCOhost databases)	Undergraduate nursing students	N = 14 studies (2007–2021)	Nil	Whittemore and Knafl (2005) framework	i) Nursing students’ breastfeeding knowledge
							ii) Sources of the breastfeeding knowledge.
Boyd-Turner *et al*. 2016^13^	Qualitative: descriptive	Semi-structured interview to evaluate student experience	Tertiary hospital: women, children, and neonates	Graduates who had completed bachelor of nursing < 12 months prior (N = 6)	Nil	Constant comparative method by Glaser and Strauss (1967) and Erlandson *et al*. (1993)	i) Clinical support and student facilitation
							ii) Education opportunities and debriefing
							iii) Career planning.
Chapman *et al*. 2012^31^	Mixed methods cross-sectional survey	Questionnaire with open-ended questions	Two Western Australia tertiary institutions	Third-year undergraduate nursing students (N = 100) compared with fifth-year medical students N = 161	Nil	Spearman rank correlation coefficients	Student knowledge, attitudes, and beliefs about lesbian, gay, bisexual, and transgender parents.
						Open-ended responses coded	
Forster *et al*. 2016^32^	Qualitative: observation of simulation participation	Videotaped simulation experience	Queensland tertiary institution	Fourth-year undergraduate nurse-midwife dual-degree students (N = 6)	Two sequential simulations (30 mins):	Audiotaped debriefing transcripts analyzed using Charmaz’s approach	Participant experience of the simulation:
		Debriefing included questions to elicit student reflections on the experience			i) High-fidelity, unsuccessful neonatal resuscitation		i) Feeling unprepared
					ii) Bereaved mother.		ii) Communication changes
							iii) The value of the simulation
							iv) Personal reactions.
Gamble 2017^19^	Cohort: mixed methods	Immediate:Likert scale, Satisfaction with Simulation Experience Scale, open-/closed-ended questionnaire	Victoria tertiary institution	Third-year undergraduate nursing students enrolled in a pediatric elective unit (N = 28)	Student-led simulated ward:	Quantitative: descriptive	Immediate:
		Post-placement: open-/closed-ended questionnaire			**•** High-fidelity manikin—child with deterioration	Qualitative: thematic analysis	i) Satisfaction, debriefing and reflection, clinical reasoning, clinical learning
		Post-employment: open-/closed-ended questionnaire			**•** Medium-fidelity manikin—child with gastroenteritis		ii) Preparation for placement
					**•** 2 adolescent simulated patients—diabetes and osteomyelitis		iii) Development and/or refinement of personal attributes
					**•** 4 adult simulate patients—acted as parents.		iv) Impact on current/future curriculum.
					Students given ward-based registered nurse roles, including handover, planning and providing care, documenting, managing breaks, supporting colleagues.		Post-clinical placement:
					Expected to demonstrate:		i) Key learning and ability and success in applying this in the clinical environment
					**•** Safe and capable clinical skills		ii) Overall performance feedback.
					**•** Professional attributes		Post-employment:
					**•** Management of challenging situations.		i) Perceived impact of the simulation on their current clinical practice.
					Post debrief with faculty members.		
Goddard *et al*. 2010^33^	Qualitative: action research	Interviews, project meeting minutes, documentation, and researchers’ reflective journal	Regional community and regional tertiary institute	Final-year undergraduate nursing students studying chronic disease and disability (N = 9)	80-hour practicum placement, facilitators worked alongside students to care for 5 families of children with intellectual disabilities:	Method of Burnard (2003), Meyer (2000), and Hope and Waterman (2003)	i) Planning—parents, student nurses, project facilitators, and matching students with families
					i) Conduct a family assessment using Gordons and Calgary tools		ii) Family assessment
					ii) Identify educational strategies		iii) Goal setting
					iii) Present findings to the families and set functional goals.		iv) Health resource development
							v) Evaluation—awareness, understanding, valuing.
Miller-Rosser *et al*. 2019^34^	Qualitative: case study	Interviews, focus group, and open-ended questionnaires	Queensland tertiary institution	Second-year undergraduate nursing students (N = 102)	Kidney Kids Camp—annual 5-day camp for children living with kidney disease. Primary care clinical placement, care for children 24/7 supported by renal nurses and academics.	Thematic analysis: Braun and Clarke’s (2006) 6-step approach coded using an inductive process	i) Developing knowing through
							ii) children’s eyes
							iii) Entering the world of primary care
							iv) Facing a journey of self-discovery
							v) It’s all in the way you communicate
							vi) It makes you think differently about nursing.
Reid-Searle *et al*. 2012^4^	Cohort: convergent mixed methods design	Open-/closed-ended questionnaire	Queensland tertiary institution	Undergraduate nursing students across all years (N = 22)	Student-led medical play activities with children (2–8 years) using cloth puppets and dolls	Quantitative: Wilcoxon signed rank test	Quantitative: Pre and post confidence, clinical reasoning, clinical learning skills
					i) Handwashing—using glitter bug	Qualitative: NVivo 12 thematic analysis	Qualitative: Communication, preparation for the role and readiness for work.
					ii) Personal protective equipment		
					iii) Wound care		
					iv) Vital signs		
Yang *et al*. 2018^36^	Systematic review: student knowledge of breastfeeding	Databases: CINAHL, MEDLINE, ProQuest, PubMed, Scopus, and Cochrane	Undergraduate nursing students, medical students, and general practice registrars	N = 14 studies (2000–2017)	Nil	Critical Appraisal Skills Program tools	i) Breastfeeding knowledge and attitudes toward breastfeeding
							ii) Breastfeeding education received by students
							iii) Factors influencing breastfeeding knowledge and attitudes.

## Appendix IV: Peer-reviewed source extraction matrix

**Table:**

Evidence source	Recommendation for preparation	Preparation example	Student experiences
Bowdler *et al*. 2023^30^	Innovative breastfeeding curriculum based on the World Health Organization 10 Steps to Successful Breastfeeding links theory and practice.	Seven-week online breastfeeding module delivered within an elective pediatric unit, designed to link theory and practice using semantic wave principles.	Students described the module as engaging and flexible and reported that it strengthened their evidence-based understanding of how to support families.
Bowdler *et al*. 2022^35^	Strong theoretical framework and clinical placements to integrate students’ informal knowledge with formal education and enhance nursing students’ understanding and support of breastfeeding women and their children in clinical settings.	Integrative review: Nursing students’ breastfeeding knowledge, both informal knowledge and formal education.	**•** Personal breastfeeding experience was the key element for informal knowledge and could be positive or negative
	Improve nursing education and practice through global and standardized content for breastfeeding education and practice—World Health Organization 10 Steps to Successful Breastfeeding.		**•** Breastfeeding knowledge increased through formal education
			**•** Clinical placement valuable to gain hands-on experience
			**•** Superficial learning may not translate into practice.
Boyd-Turner *et al*. 2016^13^	An approachable and supportive clinical facilitator, educational opportunities, and debriefing provided positive experiences on placement.	Supported pediatric clinical placement with approachable clinical facilitators, educational opportunities, and structured debriefing.	Students described the placement as positive and supportive, and some reported that it influenced their future employment choices.
Chapman *et al*. 2012^31^	Health professionals caring/managing children of lesbian, gay, bisexual, and transgender parents need:	Educational preparation focused on inclusive care for children of lesbian, gay, bisexual, and transgender parents, including awareness-building and respectful, nonjudgmental approaches to family diversity.	Nursing students reported lower perceived knowledge than medical students regarding care for families with diverse genders and sexualities.
	i) To be open-minded, nonjudgmental, and respectful		
	ii) To increase awareness and understanding through educational interventions		
	iii) To provide resources and support systems for themselves, parents, and patients.		
Forster *et al*. 2016^32^	Simulation is valuable to prepare students for a bereavement situation.	Neonatal resuscitation simulation where students interact with high-fidelity neonatal resuscitation (manikin), followed by an interaction with the bereaved parent.	Students:
			**•** Felt unprepared and challenged by communication within team and with family
			**•** Recognized the value of confidence in providing effective communication
			**•** Identified personal responses to traumatic situations.
Gamble 2017^19^	Multisource evaluation clearly indicates the value of simulation for student placement preparation and confidence.	Simulated complex pediatric ward for a day.	Students:
	Inclusion of complex ward simulation in the curriculum should be encouraged as it facilitates students’ exposure to a typical ward in a safe patient environment.		**•** Increased confidence and competence
			**•** Developed clinical reasoning and clinical learning
			**•** Developed professional attributes, including teamwork, critical thinking, decision-making, time management and prioritization skills, and communication skills with children
			**•** Were able to apply key learnings from simulation in the clinical environment through responsibility to care for 4 children from early period of placement.
Goddard *et al*. 2010^33^	University-initiated, community-focused professional experience placement assists in achieving the competency-based learning outcomes required for undergraduate nursing students.	Community-based alternative placement in which students worked with families of children with intellectual disability, completed family assessments, identified educational strategies, and set goals with families. Students supported by academics.	Students reported increased awareness of family needs, greater appreciation of primary care, stronger rapport with families, and improved understanding of partnership in care.
Miller-Rosser *et al*. 2019^34^	Primary care clinical placements for nursing students and partnership between the university and industry improves nursing workforce preparation.	Alternative placement experience, Kidney Camp, working in collaboration with nurse experts and academics to provide care 24/7.	Experience in the primary care setting:
			**•** Assisted students to link knowledge
			**•** Self-discovery
			**•** Recognize the challenge of communication
			**•** Value the primary care setting.
Reid-Searle *et al*. 2012^4^	Mock pediatric hospital experience is beneficial for undergraduate nursing students.	Mock pediatric hospital ward using student-led play-based activities with children aged 2–8 years.	Students reported improved confidence, clinical reasoning, and readiness to care for children, while also describing nervousness and awkwardness when they lacked prior experience with children.
Yang *et al*. 2018^36^	Nursing students should receive education on breastfeeding to prepare them to work in the area to support breastfeeding women and their children.	Breastfeeding education:	Highlighted breastfeeding knowledge gap:
	Improve breastfeeding education in undergraduate nursing curriculum through a range of formats and educational strategies	**•** Standard curriculum: maternal and child health subject and clinical placement at a hospital-based maternity unit.	**•** Knowledge about how breastfeeding benefits baby and has cost benefits for families and society
	Implement the World Health Organization 10 Steps to Successful Breastfeeding.	**•** Specific breastfeeding education interventions: adapting from the Baby Friendly Hospital Initiative 20-h breastfeeding curriculum.	**•** Limited knowledge of breastfeeding physiology and management
			Increased nursing students’ knowledge and confidence in managing breastfeeding and lactation problems.
			Limited education before clinical placement affected the students’ ability to support breastfeeding.
			Inadequate ongoing education meant supervising health professionals did not appropriately support and mentor students.
			Traditions and sociocultural experience influenced nursing students’ positive attitudes toward breastfeeding, which were crucial for promoting breastfeeding initiation and for supporting breastfeeding.

## Appendix V: Advanced Google search extraction matrix

**Table:**

Evidence source	Title	Type of evidence source	Recommendation for preparation	Preparation example	Experiences
University of South Australia 2023^37^	Bachelor of Nursing Medicine Administration and Scope of Practice Guidelines: A Resource for Students, Clinical Facilitators and Placement Providers	Guidelines	Pediatric medication:	—	—
			Students unable to act as double-checkers for pediatric medication		
			Students may function as the third person		
			Students may administer the medication if they performed the third check, were present during the medication preparation, and are directly supervised by a registered nurse during medication administration.		
Anonymous 2015^38^	A Year in Paediatric Nursing	Personal reflection	—	—	Loving the experience of placement in a pediatric medical ward and discovering a passion for pediatric nursing.
					Value of compassion, empathy, building trust with patients and their families, and going above clinical responsibilities.

## Appendix VI: Website extraction matrix

**Table:**

sAuthor	Type of evidence source	Recommendation for preparation	Preparation example	Outcome of preparation
Frommolt and Rands 2014^39^	ACCYPN conference abstract	Alternative placement experiences offer immersion with continuous support from academic supervisors.	International community placement in which final-year students worked with academic supervisors to promote health and conduct assessments for children and families.	Students were reported to develop confidence, improved understanding of families, and personal skills through immersion and academic support.
Colgrave and Miller-Rosser 2016^40^	ACCYPN conference abstract	Alternative placement experiences offer unique and valuable experiences with continuous support from academic supervisors.	Community placement involving interaction with children with chronic kidney disease and their families.	Students described a more positive view of pediatric care, greater recognition of care complexity, and stronger awareness of teamwork and the impact of chronic illness on families.
ACCYPN 2018^41^	Position statement:	ACCYPN minimum standards used for curriculum design and assessment in undergraduate education	—	—
	Nurses are crucial in providing health care to children, young people, and their families across various settings	Clinical education is an integral part of the educational program		
	Education and continuous professional development affect the quality of care delivered	Clinical experience supervised by registered nurses with specialist qualifications relevant to the practice setting.		
	It is essential for nurses to be well-prepared for both general and specialized practice.			
ACCYPN 2018^42^	Position statement:	Working with children clearance	—	—
	The minimum standards expected of all nurses (both Registered and Enrolled) working with children, young people, and their families to ensure their best possible health and psychological well-being, accomplished through meaningful collaboration in planning and delivering care.	Advocates child rights		
		Child and family-centered care		
		Ensures safety, minimizes harm		
		Age-appropriate care		
		Recognition and response (pediatric basic life support)		
		Pediatric drug calculations and safe administration		
		Child abuse recognition and mandatory reporting.		
Australian Association for Infant Mental Health 2019^43^	Competency guidelines, which address the support and education of nursing students and inform educational content.	Skills, knowledge, and attributes of infant mental health	—	—
		Clinical facilitator preparation		
		Family and communication skills		
		Mental health lifespan content		
		Multidisciplinary team		
		Professional well-being through supervision and reflection.		
World Association for Infant Mental Health 2016^44^	Position paper	Service providers should have resources for training and supervising caregivers in infant care institutions	—	—
		Educational content regarding risks to development		
		Professional well-being through supervision and reflection.		
Australian Commission on Safety and Quality in Health^1^	National standard, which defines workforce as including students.	The workforce has the necessary skills, knowledge, experience, and clinical guidelines for safe child- and family-focused health care, including pediatric guidelines in a child-safe setting. Governance must support workforce roles and responsibilities.	—	—
	Charter on the Rights of Children and Young People in Healthcare Services in Australia (the Charter)—11 rights aimed at ensuring that the care provided to children is appropriate and acceptable. Three key principles underpin the Charter:	Educational content includes:		
	i) In the child’s best interests	• Partnerships: consent, developmentally appropriate communication		
	ii) All children are to be listened to and taken seriously	• Safety: environment, child protection training, working with children check		
	iii) The family is recognized as the fundamental decision-making unit in a child’s life.	**•** Infection prevention: visitors, hygiene, antiseptics, isolation, invasive devices, antimicrobial stewardship		
		**•** Medication safety: height, weight, dosage calculations, double-checking, discharge education		
		**•** Comprehensive care: mental health assessment, trauma-informed care, end-of-life care, legal and ethical obligations		
		**•** Minimizing harm: pressure injury, falls, allergies, fluid management, child-friendly foods, intravenous/feeding tube safety, delirium, suicide/self-harm response, alternatives to coercive interventions, aggression management		
		**•** Communication: identification, procedure matching, handover		
		**•** Blood management.		
		Response to deterioration: iSoBAR NOW, PARROT charts, escalation, sepsis pathway, equipment sizes.		
NSW Government 2022^45^	Policy document	Children and adolescents must be cared for by staff with appropriate skills, experience, and qualifications to meet their physical, psychological, developmental, communication, and cultural needs.	Core training modules in:	—
			**•** Pediatric resuscitation	
			**•** Recognition of deterioration	
			**•** Child protection pediatric medication safety.	
NSW Government 2024^46^	Policy document	Outlines mandated learning for nurses working with children and their families.	—	—
NSW Government 2024^50^	Core training modules	—	Online training for mandatory requirements outlined in New South Wales policy document for nurses working with children and their families.	—
NSW Government 2024^47^	Policy document	All clinicians must understand the importance of the first 2000 days in their daily practice, whether they work with infants and young children or with adults and the elderly.	—	—
Haslam *et al*. 2023^48^	Report on the collaboration of researchers from Australia, the US, and the UK with the Social Research Centre. Data from 8503 Australians in 2021. First nationally representative estimates of the prevalence of 5 types of child maltreatment in Australia, along with their associated health outcomes over the lifespan.	Health practitioners require preservice training and ongoing education to understand, identify, and respond to child maltreatment.	—	—
Australian Nursing and Midwifery Accreditation Council 2019^24^	Accreditation standards aim to produce a mobile and generalist workforce and ensure safety for the public without the need for specialist training to care for infants and children.	The program’s content and subject learning outcomes ensure integrated knowledge of care over the lifespan and across contexts of nursing practice.	—	—
Association for the Wellbeing of Children in Healthcare 2008^49^	Professional standards aim to recognize that the medical and psychosocial needs of children and adolescents differ from those of adults, and aim to ensure that this is reflected in the health services provision of safe, quality care through appropriately trained staff.	Staff must be:	—	—
		• Trained to meet the specific needs of children and adolescents		
		• Compliant with child protection checks, annual training, criminal record checks, and review of disciplinary history		
		• Trained in pediatric life support		
		• Trained in recognizing and meeting the special needs of children and adolescents, including pediatric pain management, developmentally appropriate communication, and cultural competency		
		• Able to access pediatric pharmacopoeia, clinical practice guidelines, and a poisons helpline, with annual reviews to keep resources up to date.		

ACCYPN, Australian College of Children’s and Young Peoples Nurses; iSoBAR NOW, identify, situation, observations, background, agreed plan, read back—needs, observations, why; NSW, New South Wales; PARROT, Paediatric Acute Recognition and Response Observation Tools.

## Appendix VII: Australian pediatric hospital extraction matrix

**Table:**

Hospital	Type of information	Preparation
Sydney Children’s Hospital	Policy for students on clinical placement^51^	**•** Mandatory training modules
	https://resources.schn.health.nsw.gov.au/policies/policies/pdf/2017-190.pdf	**•** Students issued New South Wales Health employee identification and access to the New South Wales Health Learning Management System
		**•** All students complete eLearning modules before placement
		**•** Mandatory training integrated into local student orientation
		**•** Outlines preparation for clinical facilitators
Royal Children’s Hospital—Melbourne	Explicit policies and agreement for students at Royal Children’s Hospital	—
	Undergraduate student scope of practice document^52^	
	https://www.rch.org.au/uploadedFiles/Main/Content/nursing-education/Scope%20of%20Practice_July2021.pdf	
	Undergraduate clinical practice manual^53^	
	https://www.rch.org.au/uploadedFiles/Main/Content/nursing-education/clinical-placement-manual.pdf	
Mater Children’s Private Brisbane	—	—
Centenary Hospital for Women and Children—Canberra	Commitment to supporting students for workforce	—
	Clinical teaching and learning strategy document^54^	
	Canberra Health Services Clinical Learning and Teaching Strategy 2023–2027	
Perth Children’s Hospital	—	—
Queensland Children’s Hospital	—	—
Women’s and Children’s Hospital North Adelaide	—	—
Monash Children’s Hospital	—	—
Randwick Children’s Hospital	—	—

## References

[1] Australian Commission on Safety and Quality in Health Care. National safety and quality health service standards: user guide for acute and community health service organisations that provide care for children [internet]. Australian Commission on Safety and Quality in Health Care; 2018 [cited 2024 Sep 25]. Available from: https://www.safetyandquality.gov.au/sites/default/files/migrated/National-Safety-and-Quality-Health-Service-Standards-User-Guide-for-Acute-and-Community-Health-Service-Organisations-the-Provide-Care-for-Children.pdf.

[2] Russell L. Milestones in Australian nursing. In: Daly J Speedy S Jackson D, editors. Contexts of nursing: an introduction. MacLennan and Petty; 2000. pp. 11-24.

[3] Al-Omari E Dorri R Blanco M Al-Hassan M. Innovative curriculum development: embracing the concept-based approach in nursing education. Teach Learn Nurs 2024;19(4):324-33.

[4] Reid-Searl K Crowley K Anderson C Blunt N Cole R Suraweera D. A medical play experience: preparing undergraduate nursing students for clinical practice. Nurse Educ Today 2021;100:104821. 10.1016/j.nedt.2021.104821 33751997

[5] Universities Australia. More clinical placements needed to grow nursing workforce [internet]. Universities Australia; 2023 [cited 2026 Jan 14]. Available from: https://universitiesaustralia.edu.au/media-item/more-clinical-placements-needed-to-grow-nursing-workforce/.

[6] Frost KA Metcalf EP Brooks R Kinnersley P Greenwood SR Powell CV. Teaching paediatric communication skills to medical students. Adv Med Educ Pract 2015;6:35-43. 10.2147/AMEP.S68413 PMC4303365 25653569

[7] Gibson-Young L Lambert AW Yordy M Wang CH. Exploring outcomes from an innovative, paediatric-focused intervention with undergraduate nursing students. J Pediatr Nurs 2023;68:30-4. 10.1016/j.pedn.2022.09.002 PMC9621638 36328911

[8] Taylor C Foster J. The experiences of new graduate nurses working in a paediatric setting: a qualitative systematic review. J Pediatr Nurs 2022;67:e234-48. 10.1016/j.pedn.2022.07.005 35914989

[9] McKeever S Whiting L Anderson D Twycross A. Causes of ATtrition in CHIldren’s NursinG (CATCHING): an exploratory mixed method study. Nurse Educ Pract 2018;32:44-51. 10.1016/j.nepr.2018.06.012 30016755

[10] World Health Organization. Fact sheet: nursing and midwifery [internet]. WHO; 2024 [cited 2023 Jun 7]. Available from: https://www.who.int/news-room/fact-sheets/detail/nursing-and-midwifery.

[11] Sonney J Peck JL. The cost of caring during COVID-19: a clarion call to action to support the paediatric advanced practice nursing workforce. J Pediatr Health Care 2023;37(6):658-72. 10.1016/j.pedhc.2023.08.003 37725029

[12] Courtwright SE Turi E Barr EA Burns JC Gigli KH Bennett CR. Facilitators and barriers to paediatric nurse practitioner practice in the United States: a systematic review. J Pediatr Health Care 2024;38(4):520-43. 10.1016/j.pedhc.2023.12.003 PMC11222060 38284964

[13] Boyd-Turner D Bell E Russell A. The influence student placement experience can have on the employment choices of graduates: a paediatric nursing context. Nurse Educ Pract 2016;16(1):263-8. 10.1016/j.nepr.2015.10.001 26497419

[14] Mandy El A Geraldine R. Fostering the retention of a skilled paediatric clinical workforce through undergraduate and postgraduate nurse education. J Children Young Peoples Health 2024;5(1).

[15] Betz CL. Paediatric nursing education: trends, challenges and aspirations. J Pediatr Nurs 2021;58:A7-8. 10.1016/j.pedn.2021.04.030 34053698

[16] McCarthy AM Wyatt JS. Undergraduate paediatric nursing education: issues, challenges and recommendations. J Pediatr Nurs 2014;30(2):130-8. 10.1016/j.profnurs.2013.07.003 24720941

[17] Buckley L Berta W Cleverley K Medeiros C Widger K. What is known about paediatric nurse burnout: a scoping review. Hum Resour Health 2020;18(1):9-23. 10.1186/s12960-020-0451-8 PMC7014723 32046721

[18] Missen K McKenna L Beauchamp A. Work readiness of nursing graduates: current perspectives of graduate nurse program coordinators. Contemp Nurse 2015;51(1):27-38. 10.1080/10376178.2015.1095054 26394245

[19] Gamble AS. Simulation in undergraduate paediatric nursing curriculum: evaluation of a complex ‘ward for a day’ education program. Nurse Educ Pract 2017;23:40-7. 10.1016/j.nepr.2017.02.001 28214431

[20] Borhani F Abbaszadeh A Nakhaee N Roshanzadeh M. The relationship between moral distress, professional stress, and intent to stay in the nursing profession. J Med Ethics Hist Med 2014;7:3-8. PMC4263391 25512824

[21] Bong HE. Understanding moral distress: how to decrease turnover rates of new graduate paediatric nurses. Pediatr Nurs 2019;45(3):109-14.

[22] Nursing and Midwifery Board of Australia. Registered nurse standards for practice [internet]. NMBA; 2016 [cited 2024 Sep 25]. Available from: https://www.nursingmidwiferyboard.gov.au/Codes-Guidelines-Statements/Professional-standards/registered-nurse-standards-for-practice.aspx.

[23] Clark R Locke M Bialocerkowski A. Paediatric terminology in the Australian health and health education context: a systematic review. Dev Med Child Neurol 2015;57(11):1011-8. 10.1111/dmcn.12803 25963398

[24] Australian Nursing and Midwifery Accreditation Council. Registered nurse: accreditation standards 2019 [internet]. ANMAC; 2019 [cited 2024 Sep 25]. Available from: https://anmac.org.au/sites/default/files/2024-06/registerednurseaccreditationstandards2019_0.pdf.

[25] Peters MDJ Godfrey C McInerney P Munn Z Tricco AC Khalil H. Scoping reviews. In: Aromataris E Lockwood C Porritt K Pilla B Jordan Z, editors. JBI Manual for Evidence Synthesis [internet]. JBI; 2024 [cited 2026 Aug 3]. Available from: https://synthesismanual.jbi.global.

[26] Tricco AC Lillie E Zarin W O’Brien KK Colquhoun H Levac D. PRISMA extension for scoping reviews (PRISMA-ScR): checklist and explanation. Ann Intern Med 2018;169(7):467-73. 10.7326/M18-0850 30178033

[27] Peters MDJ Marnie C Tricco AC Pollock D Munn Z Alexander L. Updated methodological guidance for the conduct of scoping reviews. JBI Evid Synth 2020;18(10):2119-26. 10.11124/JBIES-20-00167 33038124

[28] Page MJ McKenzie JE Bossuyt PM Boutron I Hoffman TC Mulrow CD. The PRISMA 2020 statement: an updated guideline for reporting systematic reviews. BMJ 2021;372:n71. 10.1136/bmj.n71 PMC8005924 33782057

[29] Pollock D Peters MDJ Khalil H McInerney P Alexander L Tricco AC. Recommendations for the extraction, analysis, and presentation of results in scoping reviews. JBI Evid Synth 2023;21(3):520-32. 10.11124/JBIES-22-00123 36081365

[30] Bowdler S Nielsen W Meedya S Salamonson Y. Applying legitimation code theory to teach breastfeeding in nurse education: a case study. Nurse Educ Pract 2023;72:103780. 10.1016/j.nepr.2023.103780 37738786

[31] Chapman R Watkins R Zappia T Nicol P Shields L. Nursing and medical students’ attitude, knowledge and beliefs regarding lesbian, gay, bisexual and transgender parents seeking health care for their children. J Clin Nurs 2012;21(7-8):938-45. 10.1111/j.1365-2702.2011.03892.x 22008095

[32] Forster EM Donovan H. Enhancing bereavement support skills using simulated neonatal resuscitation. Int J Palliat Nurs 2016;22(10):500-7. 10.12968/ijpn.2016.22.10.500 27802083

[33] Goddard L Mackey S Davidson PM. Functional clinical placements: a driver for change. Nurse Educ Today 2010;30(5):398-404. 10.1016/j.nedt.2009.09.012 19883958

[34] Miller-Rosser K Colgrave J Stockhausen L. The student experience of a ‘kidney CAMP’ as a primary care clinical placement for undergraduate nursing students. Nurse Educ Today 2019;83:104198. 10.1016/j.nedt.2019.08.016 31513987

[35] Bowdler S Nielsen W Moroney T Meedya S. What knowledge of breastfeeding do nursing students hold and what are the factors influencing this knowledge: an integrative literature review. Nurse Educ Pract 2022;64:103423. 10.1016/j.nepr.2022.103423 35987031

[36] Yang SF Salamonson Y Burns E Schmied V. Breastfeeding knowledge and attitudes of health professional students: a systematic review. Int Breastfeed J 2018;13(1):8. 10.1186/s13006-018-0153-1 PMC5819656 29483935

[37] University of South Australia. Bachelor of nursing medicine administration and scope of practice guidelines: a resource for students, clinical facilitators and placement providers [internet]. University of South Australia; 2023 [cited 2024 Jan 19]. Available from: https://lo.unisa.edu.au/pluginfile.php/4046625/mod_resource/content/2/Medicines%20Guideline_UniSA_Bachelor%20of%20Nursing%20Students%20Medicines%20Scope%20of%20Practice%20Revised%20April%202023%20Updated%20August%202023.pdf.

[38] Anonymous. A year in paediatric nursing [internet]. The Footnotes Blog; 2015 [cited 2024 Jan 19]. [No longer available online.]

[39] Frommolt V Rands H. Nursing students creating supportive environments through health promotion activities for children and their families during a primary health care placement in rural Laos [internet]. Australian College of Children and Young People’s Nurses; 2014 [cited 2024 Jan 27].

[40] Colgrave J Miller-Rosser K. Exposure of undergraduate students to the paediatric population in a community setting: an innovative clinical education model [internet]. Australian College of Children and Young People’s Nurses; 2016 [cited 2024 Jan 27]. [No longer available online.]

[41] ACCYPN Board of Directors. Position statement: nurse education [internet]. Australian College of Children and Young People’s Nurses; 2018 [cited 2024 Jan 27]. Available from: https://www.accypn.org.au/client_images/3915233.pdf.

[42] ACCYPN Board of Directors. Position statement: minimum requirement for nurses caring for children and young people [internet]. Australian College of Children and Young People’s Nurses; 2018 [cited 2024 Jan 27]. Available from: https://www.accypn.org.au/client_images/3915333.pdf.

[43] Australian Association for Infant Mental Health. Infant mental health—nurturing early social and emotional wellbeing: competency guidelines endorsement for culturally sensitive, relationship-focused practice promoting infant mental health [internet]. Australian Association for Infant Mental Health; 2019 [cited 2024 Jan 27]. Available from: https://www.aaimh.org.au/media/website_pages/resources/for-professionals/infant-mental-health-competency-guidelines/AAIMH-WA-Competency-Guidelines-2nd-Edition-November-2019.pdf.

[44] World Association for Infant Mental Health. Position paper on the rights of infants [internet]. WAIMH; 2016 [cited 2024 Jan 27]. Available from: https://perspectives.waimh.org/wp-content/uploads/sites/9/2017/05/PositionPaperRightsInfants_-May_13_2016_1-2_Perspectives_IMH_corr.pdf.

[45] New South Wales Health. Policy directive: the safety and wellbeing of children and adolescents in NSW acute health facilities [internet]. NSW Government; 2022 [cited 2024 Jan 27]. Available from: https://www1.health.nsw.gov.au/pds/ActivePDSDocuments/PD2022_053.pdf.

[46] Health Education and Training Institute. Matrix and targeting guide [internet]. NSW Government; 2024 [cited 2024 Jan 27]. Available from: https://www.heti.nsw.gov.au/education-and-training/my-health-learning/mandatory-training/matrix-and-related-documents.

[47] New South Wales Health. Policy statement: NSW implementation framework for the first 2000 days [internet]. NSW Government; 2019 [cited 2024 Jan 27]. Available from: https://www1.health.nsw.gov.au/pds/ActivePDSDocuments/PD2019_008.pdf.

[48] Haslam D Mathews B Pacella R Scott JG Finkelhor D Higgins DJ. The prevalence and impact of child maltreatment in Australia—findings from the Australian Child Maltreatment Study: brief report [internet]. Queensland University of Technology; 2023 [cited 2024 Jan 27]. Available from: https://www.acms.au/resources/the-prevalence-and-impact-of-child-maltreatment-in-australia-findings-from-acms-2023-brief-report%2F.

[49] Association for the Wellness of Children in Hospital. Standards for the care of children and adolescents in health services [internet]. Paediatrics and Child Health Division, The Royal Australasian College of Physicians; 2008 [cited 2024 Jan 27]. Available from: https://awch.org.au/wp-content/uploads/2018/12/4.-Standards_Care_Of_Children_And_Adolescents.pdf.

[50] Health Education and Training Institute. Mandatory training [internet]. NSW Government; 2021 [cited 2024 Jan 27]. Available from: https://www.heti.nsw.gov.au/education-and-training/my-health-learning/mandatory-training.

[51] Sydney Children’s Hospitals Network. Student placement policy [internet]. NSW Government; 2021 [cited 2024 Jan 27]. Available from: https://resources.schn.health.nsw.gov.au/policies/policies/pdf/2017-190.pdf.

[52] Royal Children’s Hospital. Scope of practice for student nurses: entry to practice student nurse program acute, sub-acute, community and mental health placement [internet]. Royal Children’s Hospital; 2023 [cited 2024 Jan 27]. Available from: https://www.rch.org.au/uploadedFiles/Main/Content/nursing-education/Scope%20of%20Practice_Aug23.pdf.

[53] Royal Children’s Hospital. Undergraduate nursing student clinical placement manual [internet]. RCH; 2023 [cited 2024 Jan 27]. Available from: https://www.rch.org.au/uploadedFiles/Main/Content/nursing-education/clinical-placement-manual.pdf.

[54] Canberra Health Services. Clinical learning and teaching strategy 2023–2027 [internet]. ACT Government; 2023 [cited 2024 Jan 27]. Available from: https://www.canberrahealthservices.act.gov.au/__data/assets/pdf_file/0012/2222040/85f87c3f27ab0727c260c71172d75e1a1a7eb6a8.pdf.

[55] Curtin University. Bachelor of science (nursing). Curtin University; 2023 [cited 2024 Jan 29]. Available from: https://www.curtin.edu.au/study/offering/course-ug-bachelor-of-science-nursing--b-nurs.

[56] Edith Cowan University. Bachelor of science (nursing) [internet]. Edith Cowan University; 2023 [cited 2024 Jan 29]. Available from: https://www.ecu.edu.au/degrees/courses/bachelor-of-science-nursing.

[57] Notre Dame University. Bachelor of nursing [internet]. Notre Dame University; 2023 [cited 2024 Jan 29]. Available from: https://www.notredame.edu.au/programs/school-of-nursing/undergraduate/bachelor-of-nursing.

[58] Murdoch University. Undergraduate course: bachelor of nursing [internet]. Murdoch University; 2023 [cited 2024 Jan 29]. Available from: https://www.murdoch.edu.au/course/undergraduate/b1417?_gl=1*xgvg97*_gcl_au*Mjk3NTgzMzcwLjE3OTA3MjcxNzI.*_ga*MTU5MTY2ODkzMy4xNzkwNzI3MjAw*_ga_JJL7264DX3*czE3OTA3MjcyMDAkbzEkZzEkdDE3OTA3MjcyMDAkajYwJGwwJGg2NjM1NzcxOTQ.&studentType=domestic.

[59] Western Sydney University. Bachelor of nursing [internet]. Western Sydney University; 2023 [cited 2024 Jan 29]. Available from: https://www.westernsydney.edu.au/future/study/courses/undergraduate/bachelor-of-nursing.

[60] Deakin University. Bachelor of nursing: undergraduate degree [internet]. Deakin University; 2023 [cited 2024 Jan 29]. Available from: https://www.deakin.edu.au/course/bachelor-nursing.

[61] Griffith University. Bachelor of nursing [internet]. Griffith University; 2023 [cited 2024 Jan 29]. Available from: https://www.griffith.edu.au/study/degrees/bachelor-of-nursing-1162.

[62] Queensland University of Technology. Bachelor of nursing [internet]. Queensland University of Technology; n.d. [cited 2024 Jan 29]. Available from: https://www.qut.edu.au/courses/bachelor-of-nursing.

[63] University of Queensland. Bachelor of nursing [internet]. University of Queensland; 2023 [cited 2024 Jan 29]. Available from: https://study.uq.edu.au/study-options/programs/bachelor-nursing-2241.

[64] University of Melbourne. Career pathways: how to become a nurse [internet]. University of Melbourne; 2023 [cited 2024 Jan 29]. Available from: https://study.unimelb.edu.au/find/pathways/nurse/.

[65] Schwartz S. Educating the nurse of the future: report of the independent review into nursing education [internet]. Department of Health, Commonwealth of Australia; 2019 [cited 2024 Jan 29]. Available from: https://www.health.gov.au/sites/default/files/documents/2019/12/educating-the-nurse-of-the-future.pdf.

[66] Mould JM. Pediatric nursing: an investigation of the effect of specialist pediatric nurse education on the quality of children’s nursing care in Western Australia [dissertation]. Edith Cowan University; 2012.

[67] Henry R. Review of health services for children, young people and families within the NSW health system [internet]. NSW Health; 2019 [cited 2024 Jan 27]. Available from: https://www.health.nsw.gov.au/kidsfamilies/paediatric/Documents/henry-review.pdf.

[68] Lines LE Hutton AE Grant J. Integrative review: nurses’ roles and experiences in keeping children safe. J Adv Nurs 2017;73(2):302-22. 10.1111/jan.13101 27509334

[69] Aasen L Johannessen AK Ruud Knutsen I Werner A. The work of nurses to provide good and safe services to children receiving hospital-at-home: a qualitative interview study from the perspectives of hospital nurses and physicians. J Clin Nurs 2022;31(17-18):2495-506. 10.1111/jocn.16062 34570945

[70] Lei Y-Y Zhu L Sa YTR Cui X-S. Effects of high-fidelity simulation teaching on nursing students’ knowledge, professional skills and clinical ability: a meta-analysis and systematic review. Nurse Educ Pract 2022;60:103306. 10.1016/j.nepr.2022.103306 35202957

[71] Scurlock-Evans L Upton P Rouse J Upton D. To embed or not to embed? A longitudinal study exploring the impact of curriculum design on the evidence-based practice profiles of UK pre-registration nursing students. Nurse Educ Today 2017;58:12-8. 10.1016/j.nedt.2017.07.011 28806648

[72] MacKinnon K Marcellus L Rivers J Gordon C Ryan M Butcher D. Student and educator experiences of maternal-child simulation-based learning: a systematic review of qualitative evidence. JBI Database System Rev Implement Rep 2017;15(11):2666-706. 10.11124/JBISRIR-2016-003147 29135750

[73] Cant R Ryan C Cooper S. Nursing students’ evaluation of clinical practice placements using the clinical learning environment, supervision and nurse teacher scale: a systematic review. Nurse Educ Today 2021;104:104983. 10.1016/j.nedt.2021.104983 34229233

[74] Zeydani A Atashzadeh-Shoorideh F Abdi F Hosseini M Zohari-Anboohi S Skerrett V. Effect of community-based education on undergraduate nursing students’ skills: a systematic review. BMC Nurs 2021;20(1):1-233. 10.1186/s12912-021-00755-4 PMC8600777 34794436

[75] Al Touby S. Impact of alternative practicum approach on enhancing pediatric clinical learning among male nursing students in Oman. Int J Nurs Care 2020;8(2):46.

[76] Brown J Stevens J Kermode S. Supporting student nurse professionalisation: the role of the clinical teacher. Nurse Educ Today 2012;32(5):606-10. 10.1016/j.nedt.2011.08.007 21907468

[77] Raines DA. A dedicated education unit for maternal-newborn nursing clinical education. Nurs Womens Health 2016;20(1):20-7. 10.1016/j.nwh.2015.12.005 26902437

[78] Alharbi A Nurfianti A Mullen RF McClure JD Miller WH. The effectiveness of simulation-based learning (SBL) on students’ knowledge and skills in nursing programs: a systematic review. BMC Med Educ 2024;24(1):1099. 10.1186/s12909-024-06080-z PMC11459713 39375684

